# Structural and Functional Study of Yer067w, a New Protein Involved in Yeast Metabolism Control and Drug Resistance

**DOI:** 10.1371/journal.pone.0011163

**Published:** 2010-06-17

**Authors:** Tatiana Domitrovic, Guennadi Kozlov, João Claudio Gonçalves Freire, Claudio Akio Masuda, Marcius da Silva Almeida, Mónica Montero-Lomeli, Georgia Correa Atella, Edna Matta-Camacho, Kalle Gehring, Eleonora Kurtenbach

**Affiliations:** 1 Programa de Biologia Molecular e Estrutural, Instituto de Biofísica Carlos Chagas Filho, Universidade Federal do Rio de Janeiro, Rio de Janeiro, Brazil; 2 Programa de Biologia Molecular e Biotecnologia, Instituto de Bioquímica Médica, Universidade Federal do Rio de Janeiro, Rio de Janeiro, Brazil; 3 Groupe de recherche axé sur la structure des protéines, Department of Biochemistry, McGill University, Montréal, Quebec, Canada; 4 Centro Nacional de Ressonância Magnética Nuclear, Instituto de Bioquímica Médica, Universidade Federal do Rio de Janeiro, Rio de Janeiro, Brazil; 5 Instituto Nacional de Entomologia, Conselho Nacional de Desenvolvimento Científico e Tecnológico/MCT, Rio de Janeiro, Brazil; 6 Instituto Nacional para Pesquisa Translacional em Saúde e Ambiente na Região Amazônica, Conselho Nacional de Desenvolvimento Científico e Tecnológico/MCT, Rio de Janeiro, Brazil; University of Queensland, Australia

## Abstract

The genome of *Saccharomyces cerevisiae* is arguably the best studied eukaryotic genome, and yet, it contains approximately 1000 genes that are still relatively uncharacterized. As the majority of these ORFs have no homologs with characterized sequence or protein structure, traditional sequence-based approaches cannot be applied to deduce their biological function. Here, we characterize *YER067W*, a conserved gene of unknown function that is strongly induced in response to many stress conditions and repressed in drug resistant yeast strains. Gene expression patterns of *YER067W* and its paralog *YIL057C* suggest an involvement in energy metabolism. We show that yeast lacking *YER067W* display altered levels of reserve carbohydrates and a growth deficiency in media that requires aerobic metabolism. Impaired mitochondrial function and overall reduction of ergosterol content in the *YER067W* deleted strain explained the observed 2- and 4-fold increase in resistance to the drugs fluconazole and amphotericin B, respectively. Cell fractionation and immunofluorescence microscopy revealed that Yer067w is associated with cellular membranes despite the absence of a transmembrane domain in the protein. Finally, the 1.7 Å resolution crystal structure of Yer067w shows an alpha-beta fold with low similarity to known structures and a putative functional site.

*YER067W'*s involvement with aerobic energetic metabolism suggests the assignment of the gene name *RGI1*, standing for respiratory growth induced 1. Altogether, the results shed light on a previously uncharacterized protein family and provide basis for further studies of its apparent role in energy metabolism control and drug resistance.

## Introduction

In this work, we bring new insights into the structural and functional characterization of a previously poorly annotated gene in *Saccharomyces cerevisiae*, the open reading frame (ORF) *YER067W*. This sequence belongs to a group of approximately 1000 genes from *S. cerevisiae* (20% of the genome) that have been missed by the genome-wide methods currently used to address protein/gene function such as interactomes, phenotype screenings and homology-based functional annotation. Like the majority of uncharacterized genes from *S. cerevisiae*, *YER067W* codes for a short protein (≤20 kDa) that is present in duplicate in the yeast genome (shares 70% identity with the ORF *YIL057C*), and its homologs are restricted to fungi and contain no characterized functional domains [1].

The functional annotation of this subset of genes is particularly challenging because almost nothing can be learned from sequence comparisons. Moreover, most of the uncharacterized proteins have no tridimensional structure available, hindering a structure-based analysis. Functional hints are generally extracted from high-throughput studies performed on *S. cerevisiae*. These data are well-organized and accessible through the *Saccharomyces* Genome Database (SGD) [2], a public database that curates the yeast genes and also compiles Gene Ontology (GO) annotations, publications, interactions, and a multitude of additional information. Almost every gene has some published data compiled; however, the information is incomplete for many ORFs to define at least one aspect of the Gene Ontology (GO), which is subdivided into three major branches: Molecular function, Biological process and Cellular component. For these genes, the GO annotation awaits an experimental validation of functional hypothesis raised from high-throughput data analysis.

*YER067W* attracted our attention because of its high levels of expression in a wide range of conditions, such as intracellular iron depletion [3], carbon source restriction [4], high temperature, high osmotic stress, cold stress [5], unfolded protein response [6], and high hydrostatic pressure [7]. These data point to the importance of *YER067W* as a stress-related gene in non-standard laboratory growth conditions.

Another notable feature of *YER067W* gene regulation concerns its apparent correlation with the development of a drug-resistant phenotype in yeast. A study of *S. cerevisiae* showed that cells chronically treated with different antifungal drugs from the azole class display a consistent down-regulation of *YER067W* [8]. Furthermore, two other independent works that employed microarray analysis to address differences in gene expression between fluconazole-resistant and -susceptible strains showed that both *YER067W* and its *Candida albicans* ortholog *IPF20056* (ORF 191354) are repressed in the fluconazole-resistant strain. This trait was observed in an experimentally induced resistant strain of *S. cerevisiae* that was selected for fluconazole tolerance after growth for 400 generations in the presence of the drug [9] and was also observed in three different clinical isolates of *Candida albicans* that developed fluconazole resistance [10]. Fungal infections are particularly complex to treat because there are few classes of antifungal drugs available and the repetition and lengthy duration of treatment favors the development of drug-resistant strains [11]. The most widely used drugs belong to the azole class, which inhibit the enzyme lanosterol demethylase (Erg11), involved in the ergosterol biosynthetic pathway. Ergosterol is also the target for polyene drugs such as amphothericin B and nystatin. As both classes of drugs act on components of the same pathway, the mechanisms of resistance developed against one kind of drug are frequently effective against the other. Moreover, due to the similarity of the cellular machinery of fungi and metazoans, it is difficult to develop fungal-specific drugs [12]. Therefore, the characterization of new fungal-specific proteins involved in the development of drug resistance is of outstanding interest.

Despite the notable induction of *YER067W* in several environmental conditions, very little data concerning protein-protein or genetic interaction is available for Yer067w. Two-hybrid experiments and mass spectrometry-based identification of Yer067w interaction partners offer some potential hits, but they are never confirmed within the experiment (no more than a single identification event) or between experiments (coincident hits in different experiments) [13], [14]. Moreover, the putative interaction partners are of very diverse functions and have promiscuous interaction profiles; for example, Rpp0, a ribosomal protein that was identified as a putative Yer067w interaction partner, appears to be physically associated to more than 100 proteins of varied functions [14].

The only GO annotation available for Yer067w comes from a genome-wide localization study using GFP-tagged proteins and points to a nuclear and cytoplasmic localization for Yer067w during normal growth conditions [15].

In this work, we use existing gene regulation data and apply different experimental methodologies to improve the functional annotation of *YER067W*. We determine whether *YER067W* is adaptively important to *S. cerevisiae* and uncover the biological processes in which this stress-induced protein plays a role, including confirmation of the drug-resistance connection suggested by gene regulation data. We more accurately determine the cellular distribution of this protein by observing the localization of an untagged Yer067w protein. Finally, we present the three-dimensional structure of Yer067w and determine whether it resembles any previously characterized folds.

## Results and Discussion

### Phylogenetic analysis reveals that Yer067w is under strong selective constraint

**Figure 1:**
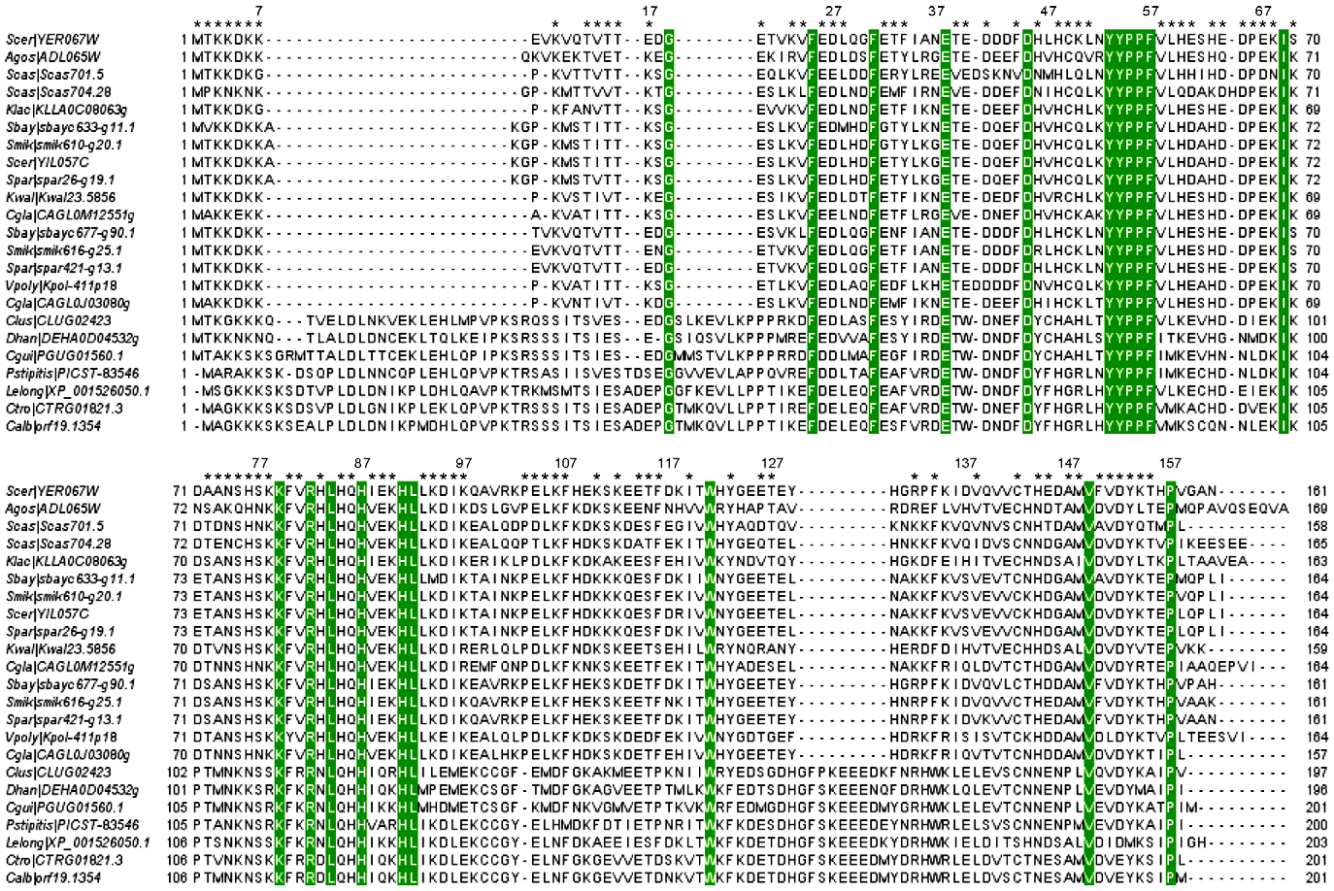
Sequence alignment of 23 proteins homologous to Yer067w protein. Sequences were identified by a PSI-BLAST search against the NR protein database and against the Fungal Orthogroups Repository [17]. Sequences are indicated with abbreviations corresponding to the species of origin, the gene identification code, and the protein amino acid number. The species and NCBI accession number, when available, are as follows: Scer, *Saccharomyces cerevisiae, YER067W*, NP_010990.1, *YIL057C*, NP_012207.1; Agos, *Ashbya gossypii, ADL065Wp*, NP_984031.1; Scas, *Saccharomyces castelli*; Klac, *Kluyveromyces lactis, KLLA0C08063g*, XP_452559.1; Sbay, *Saccharomyces bayanus*; Smik, *Saccharomyces mikatae*; Spar, *Saccharomyces paradoxus*; Kwal, *Kluyveromyces waltii*; Cgla, *Candida glabrata, CAGL0J03080g*, XP_447838.1, *CAGL0M12551g*, XP_449888.1; Vpoly, *Vanderwaltozyma (Kluyveromyces) polysporus, Kpol_411p18*, XP_001642931.1h; Clus, *Candida lusitaniae*; Dhan, *Debaryomyces hansenii, DEHA0D04532g,* XP_458633.1; Cgui, *Candida guilliermondii, PGUG_01560*, XP_001485889.1; Pstip, *Pichia stipitis, PICST_83546,* XP_001384498.1; Lelong, *Lodderomyces elongosporus, XP_001526050.1*; Ctro, *Candida tropicali*; and Calb, *Candida albicans,* CaO19.8934, *XP_710246.1.* Positions that are invariant across all species are highlighted by green boxes, and positions at which 50% or more sequences have identical residues are indicated by asterisks.

To identify Yer067w homologs, we performed BLAST and PSI-BLAST similarity searches using the Yer067w protein sequence to query the non-redundant protein database (NCBI). Despite the enhanced sensitivity of PSI-BLAST in searches for distantly-related proteins [16], both programs retrieved the same proteins, including the *S. cerevisiae* paralog Yil057c and orthologs from different fungal genera (*Candida*, *Pichia*, *Ashbya*, *Kluyveromyces*, *Lodderomyces*, *Debaryomyces,* and *Vanderwaltozyma*). The search was completed by exploring the Fungal Orthogroups Repository [17], which retrieved homologs from other *Saccharomyces* and *Candida* species (Figure 1). This analysis clearly showed that the 23 proteins homologous to Yer067w are restricted to ascomycetes belonging to the Saccharomycotina subphyla. A sequence alignment of the Yer067w family (Figure 1) reveals a high degree of conservation throughout the Saccharomycotina. A significant fraction of positions are identical in 50% or more of Yer067w family members (asterisks) and more than 20 positions are identical amongst all family members (green boxes). The limited phylogenic distribution of Yer067w homologs is characteristic of orthologous “ORFans”, genes that are typically present in only a few, generally closely related organisms and have no annotated function [18].

Many orphan genes demonstrate a higher evolving rate than non-orphan genes, changing so quickly that sequence similarity cannot be traced beyond a certain evolutionary distance. This trend suggests that those genes are under low selective pressure and perform accessory or redundant functions [19]. To evaluate if this would be the case for *YER067W*, we analyzed the evolution rate of this gene and examined the phylogenetic history of Yer067w protein family.

Evolution rate can be inferred from the ratio of the number of non-synonymous substitutions per non-synonymous site (dN) to the number of synonymous substitutions per synonymous site (dS), a measure that distinguishes the amino acid selection pressure from the background nucleotide mutation rate [20]. In a previous work analyzing dN/dS ratio in four closely related yeast species (*S. cerevisiae, S. mikatae, S. bayanus,* and *S. paradoxus*), the dN/dS ratio for *YER067W* was 0.03, indicating a relatively slow mutation rate given that the whole genome mean for dN/dS ratios in *S. cerevisiae* was 0.10 [21]. This analysis indicates that the *YER067W* locus is under strong selective constraint and that it is not a quickly evolving gene.

Moreover, the phylogenetic tree constructed using *YER067W* homologs (Figure S1) closely resembles the evolutionary history of the Saccharomycotina subphyla [22], reinforcing the conservation of this gene family. Another interesting feature of the *YER067W* family is the presence of a duplicated pair of homologs in almost every species that diverged after a whole genome duplication (WGD) event [22]. The conservation of both genes in WGD yeast species is a remarkable feature because nearly 90% of the duplicated genes in yeast genomes have lost one member of the pair in the present-day set [23]. Because truly redundant genes are unlikely to exist, the presence of two copies may be explained by a neofunctionalization, in which one duplicate evolves a useful new function while the other one performs the ancestral function, or a subfunctionalization, in which the duplicates partition ancestral functions between themselves so that both duplicates are required for full fitness (for example, if the duplicate copies become differently expressed or localized within the cell). In the following sections, this question will be addressed by analyzing gene expression regulation and phenotypes associated with deletion of *YER067W* and its paralog *YIL057C*.

### *YER067W* and *YIL057C* display different induction patterns linked to genes involved in energy metabolism

Analysis of global gene expression in several environmental conditions revealed an interesting feature of genome regulation: genes involved in common pathways or metabolic processes tend to be coordinately expressed/repressed. Therefore, the functions of characterized genes in a given regulatory cluster can suggest hypothetical functions for uncharacterized genes in the same cluster [5]. As *YER067W* expression is affected by a wide range of conditions, this approach can identify in which aspects of yeast physiology *YER067W* is involved.

By comparing several data sets from microarray experiments using the program SPELL [24], we verified that the expression of the *YER067W* gene is tightly linked to genes involved in carbohydrate metabolic processes (p value  = 5.22 10^−5^), including glycogen synthases (*GSY1* and *GSY2*) and trehalose synthase subunits (*TPS2* and *TSL1*) (Table S1). However, the paralog *YIL057C* is mainly co-regulated with genes that are involved in lipid oxidation in the peroxisome such as *PXA1*, *CTA1*, *POT1* and *ECI1* (p-value of 5.9510^−3^) (Table S1).

These results are corroborated by the differences in the promoter structures of *YER067W* and *YIL057C* as determined by the program Yeastract (Table S2) [25]. The *YER067W* promoter contains several binding sites for the stress response transcription factors Msn2/4 and Hsf1, and these sites have been functionally validated [5], [26]. Transcriptional control of *YER067W* by the main stress-responsive gene activators of *Saccharomyces cerevisiae* explains the wide range of conditions in which expression of this gene is induced. However, the co-regulation of *YER067W* with metabolism-related genes and not heat-shock proteins, which are involved with protein homeostasis, suggests that *YER067W* is mainly implicated in energetic metabolism adjustments in response to adverse conditions.

Conversely, *YIL057C* does not possess stress response elements such as Msn2/4 or Hsf1 in the promoter region (Table S2); instead, its induction seems to be governed by Adr1, a transcription factor that activates genes involved in the utilization of non-fermentable carbon sources, including many peroxisomal genes [27]. This observation is in agreement with *YIL057C* up-regulation in situations that require aerobic energetic metabolism pathways [28]–[30] and is in accordance with the SPELL results (Table S1).

### *YER067W* and *YIL057C* are important for cell growth in non-fermentable carbon sources

**Figure 2:**
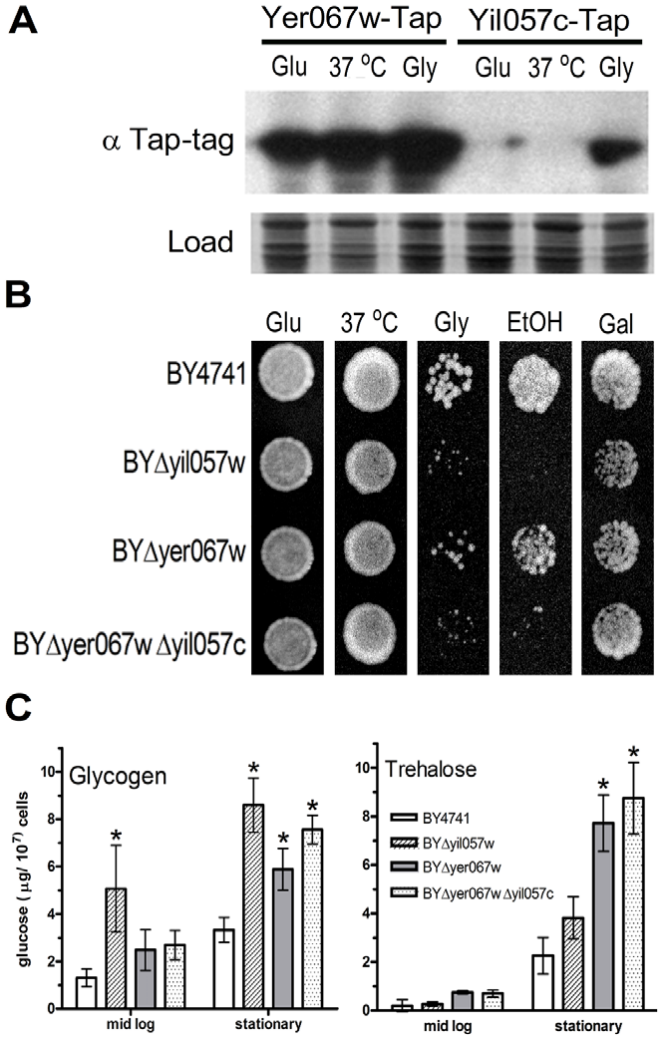
Protein expression analysis and phenotypes associated with the deletion of the *YER067W* gene. (A) Western blot of *S. cerevisiae* strain BY4741 harboring either *YER067W* or *YIL057C* genes fused to the TAP-tag reveals that these proteins are differently regulated. Cells were grown on glucose to the first log phase (Glu), heat shocked at 37°C for one hour (37°C) and grown on glycerol (Gly). The specific signal was revealed using anti-TAP antibody. As a loading control (Load), the PVDF membrane was stained with Coomassie-blue R. (B) Qualitative growth assay comparing cell growth of yeast strains deleted for *YER067W*, *YIL057C* or both and the wild type strain BY4741 grown in different media. Cells were grown to stationary phase and normalized to 10^7^ cells/mL. Serial dilutions of the suspension were spotted onto YP supplemented with 2% of glucose (Glu), galactose (Gal), glycerol (Gly) or ethanol (EtOH). All plates were grown for 28°C or 37°C for 48 hours and then photographed. Figure 2B corresponds to the third dilution step. (C) Quantification of intracellular levels of glycogen and trehalose of strains BY4741, BYΔyer067w, BYΔyil057c and the double mutant grown in YPD to either mid-log or stationary phase. Error bars indicate standard deviation from three independent experiments. The significant difference between each mutant and BY4741 is denoted with an asterisk (P<0.05 T-test).

To test if the data from global transcriptional analyses correlate with expression at the protein level, we examined the expression of TAP-tagged versions of *YER067W* or *YIL057C* genes in different growth conditions (Figure 2A). Yer067w was expressed in the presence of glucose and showed high levels of expression after heat shock at 37°C for 1 hour and when grown in glycerol, a respiratory carbon source. However, Yil057c signal was barely detectable during glucose growth and after heat shock but was clearly induced in the presence of glycerol (Figure 2A).

The strong correlation between Yil057c and Yer067w expression in the presence of non-fermentative substrates prompted us to test whether the BYΔyer067w and BYΔyil057c deletion mutant strains would present any fitness defect when grown in these type of carbon sources (Figure 2B). The mutant strains exhibited normal growth in fermentative substrates such as glucose and galactose but were clearly deficient in media containing glycerol and ethanol, in which oxidative phosphorylation is necessary to fully metabolize these substrates (Figure 2B). The BYΔyer067w strain displayed a growth delay when compared to the parental strain BY4741, while BYΔyil057c exhibited a more intense phenotype. These results correlate well with the specific induction of *YIL057C* in low glucose media (Figure 2A). The double mutant BYΔyer067wΔyil057c displayed a phenotype similar to BYΔyil057c. This kind of genetic interaction, in which the double mutant is as viable as any of the single mutants, usually indicates that the pair of proteins belongs to the same complex and/or operates in the same pathway [31]. However, the clear phenotype observed in both single mutants suggested that, despite the high degree of similarity, these ORFs may perform analogous but not fully redundant functions. Therefore, the maintenance of this pair of duplicated genes may be due to subfunctionalization of each copy.

Interestingly, neither BYΔyer067w nor BYΔyil057c differed from the wild type strain (WT) in growth in glucose-containing media at 37°C (Figure 2B). Furthermore, the mutations did not affect cell survival after high hydrostatic pressure treatment (data not shown), another stress condition that leads to *YER067W* gene induction [7].

As a next step, we quantified the intracellular content of glycogen in the mutant strains. Figure 2C shows the glycogen content in yeast cells at mid-log and stationary phases. In agreement with a previous screen for mutations that disturb glycogen metabolism [32], deletion of *YIL057C* leads to an enhanced glycogen accumulation in growing cells, while deletion of *YER067W* shows no phenotype in this situation. However, in the stationary phase, when glycogen synthesis is favored even in WT cells [33], all mutants clearly display a glycogen over-production phenotype in comparison to BY4741. *S. cerevisiae* cells store glucose as glycogen and also as trehalose [33]. To verify if the mutations were specifically affecting glycogen metabolism, we determined the trehalose content of cells from each strain. Trehalose was over-produced in the strain lacking Yer067w and, to a lesser extent, Yil057c (Figure 2C), suggesting that the lack of these proteins induce a metabolism shift toward the accumulation of reserve carbohydrates upon starvation.

Altogether, these results show that both the Yer067w and Yil057c proteins are involved in the control of energetic metabolism and significantly contribute to cell fitness, especially under respiratory growth conditions. These data assist in assignment of biological process for both ORFs in the *Saccharomyces* Genome Database (SGD). Furthermore, based on our experimental data, we propose the gene names *RGI1* and *RGI2,* as in “Respiratory Growth Induced”, for the ORFs *YER067W* and *YIL057C,* respectively.

### *YER067W* deletion enhances *S. cerevisiae* tolerance to fluconazole and amphotericin B

**Figure 3:**
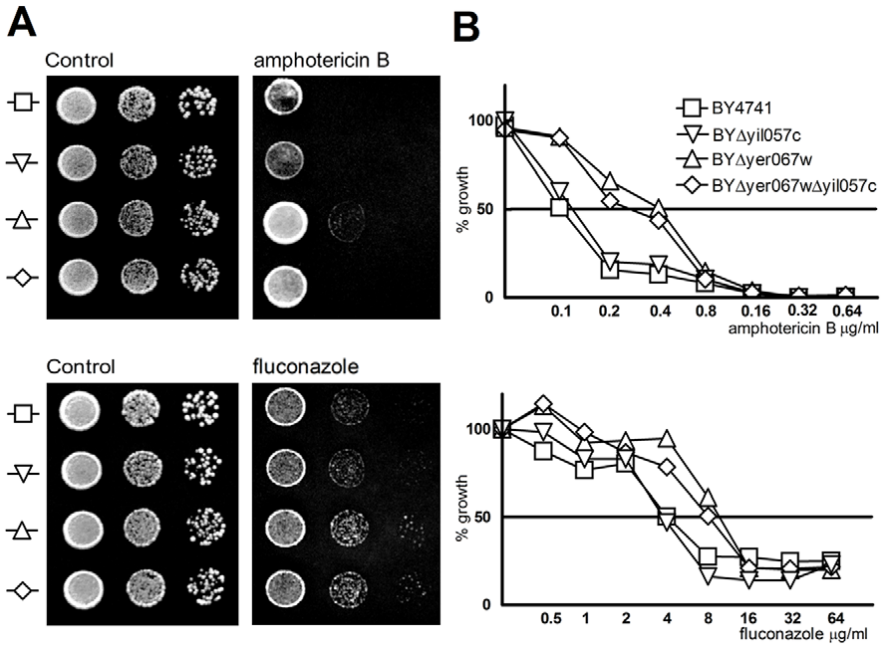
Fluconazol and amphotericin B susceptibility assay of yeast cells deleted for *YER067W* or *YIL057C* or both genes in comparison to the parental strain. (A) Qualitative assay showing BYΔYer067w drug resistance phenotype. Cells were grown up to the stationary phase and normalized to 10^7^ cells/mL. Serial dilutions of the suspension were spotted onto YPD (control) or YPD containing 16.0 µg/mL of fluconazol and 1.0 µg/mL of amphotericin B. Plates were incubated for 48 hours at 28°C, and then photographed. (B) For a quantitative drug resistance analysis, yeast cells were grown in increasing drug concentration for 16 hours, at which point the OD_600nm_ was determined. Complete (100%) growth was defined by the OD reached by the drug-free control for each strain. Standard errors are smaller than the graph symbols.

To follow up on earlier reports of Yer067w's role in drug resistance, we tested whether the strains lacking Yer067w would display a drug-resistance phenotype, as was suggested by the down-regulation of this gene in fluconazole-resistant strains [9], [10]. Cells were serially diluted and spotted on plain YPD plates and YPD plates containing 1.0 µg/mL amphotericin B or 16.0 µg/mL fluconazole (Figure 3A). In the presence of both drugs, cell colonies from BYΔyer067w and the double mutant could be detected in dilutions steps in which BY4741 and BYΔyil057c were no longer able to grow. This result points to an enhanced antifungal drug tolerance induced by *YER067W* deletion.

The resistance level was further evaluated by Minimal Inhibitory Concentration (MIC) determination using the microdilution method (Figure 3B). *YER067W* deletion promoted a slight increase in fluconazole tolerance. The MIC_50_ for BYΔyer067w and the double mutant was 8 µg/ml, while the values for BY4741 and BYΔyil057c were around 4 µg/ml. The protective effect of Yer067w deletion against treatment with the cytotoxic drug amphotericin B was stronger than observed for fluconazole, with a increase of at least 4 times when compared to the wild type strain (Figure 3B).

Classically, resistance to polyenes and azoles is achieved by an increase in the expression of multidrug transporters in the plasma membrane or modulation of the ergosterol metabolism genes [12]. Additionally, the petite phenotype, observed in cells lacking mitochondrial DNA (Rho-) and therefore, unable to respire, is associated with fluconazole resistance in different yeast species [34], [35].

**Figure 4:**
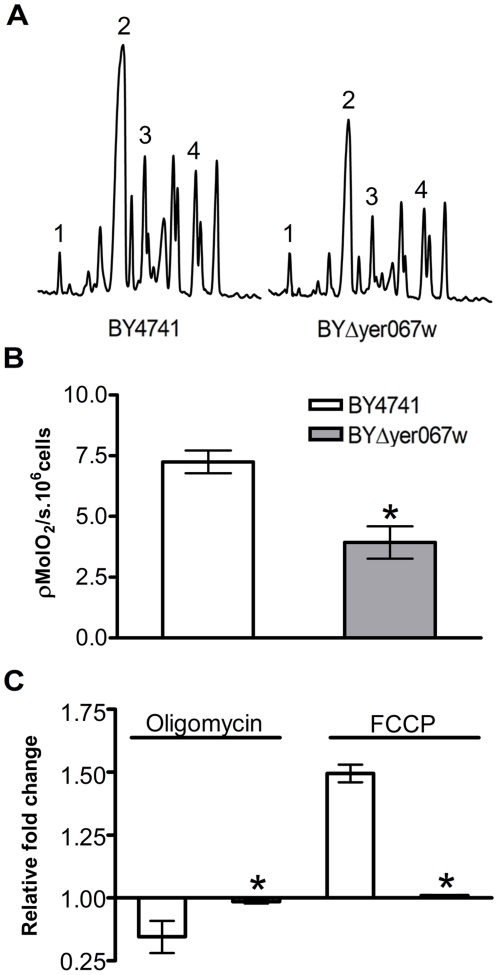
Analysis of the sterol composition and oxygen consumption of yeast cells deleted for *YER067W* gene. (A) The accumulation profile of GC sterols for BY4741 and BYΔyer067w. Peak 1, cholesterol (exogenous standard); peak 2, ergosterol; peak 3, fecosterol; and peak 4, lanosterol. Note that BYΔyer067w shows a reduced content of ergosterol (46%±5.0% four idependent extractions) indicating a down regulation in ergosterol synthetic pathway (B) Oxygen consumption rates of yeast cells growing in glycerol reveal an impaired respiratory metabolism in BYΔyer067w. (C) Relative changes in oxygen consumption rate caused by sequential addition of 5 µg/mL of oligomycin and 1 µM of FCCP. BYΔyer067w insensitivity to the drugs tested suggests that mitochondrial physiology is affected by the absence of *YER067W* protein. Asterisks indicate a significant difference from BY4741 (P<0.05 T-test).

No differences in drug resistance between BY4741 and the mutants were evident when yeast cells were grown in the presence of cycloheximide, a drug that blocks protein synthesis (data not shown). This observation indicates that the resistance conferred by a *YER067W* deletion is not due to a general effect, as expected for an over-expression of multidrug transport proteins, but is specifically related to anti-fungal drugs targeting the ergosterol metabolism. Therefore, we compared the sterol content of BYΔyer067w and WT (Figure 4A). Even though the same biosynthetic intermediates were found in both cell populations after separating different sterol species using gas mass chromatography, a consistent reduction of 46.0% of the ergosterol (SD±5.0%, four independent analysis) content in BYΔyer067w was observed (Figure 4A, peak 2).

Moreover, BYΔyer067w oxygen consumption was analyzed using a high-resolution oxygraphic system (Figure 4B and C). In agreement with the growth defect under non-fermentable carbon sources, cells lacking *YER067W* consumed approximately 50% less oxygen in comparison to WT (Figure 4B). Interestingly, the mutant cells oxygen consumption was insensitive to the addition of oligomycin, a drug that blocks the proton channel of the mitochondrial ATP synthase, leading to a reduction in oxygen consumption, and also FCCP, an ionophore that uncouples the oxidative phosphorylation from ATP synthesis and results in enhanced oxygen consumption. These results suggest that mitochondrial physiology is also affected by deletion of *YER067W*.

The overall reduction of ergosterol synthesis in BYΔyer067w could diminish the accumulation rate of the toxic intermediate 14 α-methylfecosterol upon *ERG11* inhibition by azoles and could reduce polyene binding to the membrane. This possibility could explain the resistance observed in the presence of both fluconazole and amphotericin B. However, the apparent mitochondrial dysfunction can also account for the resistance, as has been reported for yeast strains with aerobic metabolism blockage [34], [36].

It is known that the ergosterol and aerobic metabolism are tightly linked, but the mechanisms underlying this connection are not well established. For example, yeast cells deficient in ergosterol biosynthesis are known to display aberrant mitochondrial morphology and impaired respiration [28]. The reverse is also true: cells with mitochondrial defects show modulation in ergosterol gene expression and their sterol profile [34]. Presently, we cannot distinguish between these possibilities in relation to our results.

It is also important to note that the increase in drug resistance promoted by *YER067W* deletion alone is modest in comparison to the MICs of the resistant strains of *S. cerevisiae* or *C. albicans*, in which down-regulation of this gene was observed [8]–[10]. However, those strains all possess mutations in ergosterol biosynthesis genes or exhibit over-expression of multidrug transporters, alterations that are frequently associated with high degrees of drug resistance. In this scenario, it seems that *YER067W* suppression is an adaptive event that favors the fixation of a drug tolerance phenotype by inducing a series of metabolic alterations that help support growth in the presence of azoles and polyenes.

### Yer067w is associated with membranes

Cellular component is another aspect of the GO description and refers to the protein localization within the cell. The data available for Yer067w were obtained in a global study using a library of GFP-tagged *S. cerevisiae* strains [15]. To revise this information, we developed a polyclonal antibody specific to Yer067w (no cross reaction with Yil057c, data not shown) that was raised against the purified full-length Yer067w expressed in *Escherichia coli*. This new tool dispenses the need to tag an 18.9 kDa protein with larger GFP (26.9 kDa) or TAP–tag (20.2 kDa) and, therefore, prevents the risk of protein mislocalization.

**Figure 5:**
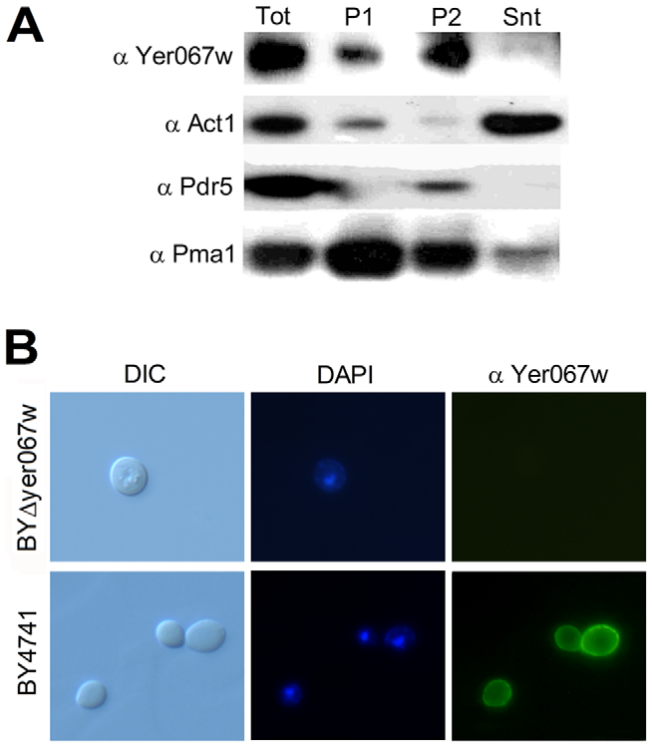
Localization analysis of Yer067w. (A) Western blot analysis of cell fractionation by differential centrifugation reveals that Yer067w is localized in cell membranes. Total protein extract was obtained from BY4741 grown overnight in glycerol. Tot, total protein extract; P1, fraction sedimented after 12,000 g centrifugation; P2, fraction sedimented after 100,000 g centrifugation; and Snt, soluble fraction collected from 100,000 g centrifugation step. (B) Immunolocalization of Yer067w. Images were collected from wild type BY4741 and the BYΔyer067w strain. Yer067w specific signal was visualized using anti-Yer067w primary antibody and 488-ALEXA conjugated secondary antibodies. DAPI-stained DNA (blue) signals and Nomarski (DIC) images were also recorded. The exposure time used for image acquisition was equal for both strains.

As a first approach to confirm the cytoplasmic and nuclear localization previously suggested for Yer067w [15], we performed a differential centrifugation cell fractionation experiment. Figure 5A shows the partition of Yer067w within the centrifugation steps using western blot analysis. As a control, we also analyzed the fractionation pattern of proteins known to localize in the cytoplasm (actin, Act1) and membranes (plasma membranes proton-ATPase, Pma1 and the multidrug transporter, Pdr5). Unexpectedly, Yer067w was concentrated in the 100,000 g sedimented fraction (P2) and, to a lesser extent, in the 12,000 g sediment (P1). Almost no signal could be detected in the cytoplasm (Snt, soluble fraction). The P1 fraction is enriched in moderately dense organelles, such as mitochondria and peroxisomes, and also contains plasma membrane fragments. The P2 fraction concentrates the low density Golgi vesicles, secretory vesicles and plasma membrane [37]. The Yer067w sedimentation pattern was the same observed for membrane bound proteins Pdr5 and Pma1, but not for Act1, which was mainly present in the soluble fraction. This result was not due to Yer067w protein aggregation and precipitation in the fractioning buffer because the recombinant Yer067w was fully soluble after 100,000 g centrifugation in this buffer condition (not shown).

To confirm the Yer067w association to membranes, localization was also checked by indirect immunofluorescence microscopy (Figure 5B). We observed a peripheral signal with a punctuate pattern, suggesting a possible lipid raft association. As the primary sequence indicates that Yer067w is a relatively charged protein with no transmembrane domains, it is possible that the interaction is driven by interactions with yet unidentified membrane-bound protein or via electrostatic interactions with membrane lipids. This localization profile clearly conflicts with the GFP data from Ghaemmaghami, *et al* [14] and adds a new perspective to Yer067w characterization.

### The crystal structure of Yer067w reveals a novel fold and a conserved putative functional site

Because proteins from the Yer067w family lie beyond homology modeling distance from any other protein with known structure or function, the structural study of Yer067w may provide valuable information for the functional characterization of this group. The initial crystallization trials with the full length recombinant Yer067w produced small, poorly diffracting, two-dimensional crystals, despite the good overall fold observed in the 2D [^1^H-^15^N] HSQC spectra (Figure S2). It is likely that the crystal formation was hindered by a highly charged, flexible N-terminus (Figure 1). Therefore, we designed a shorter construct of Yer067w, named _11V-161N_Yer067w, by removing ten N-terminal residues (1MTKKDKKEVK10). The NMR analysis of the truncated protein resulted in the same pattern of chemical shifts compared to the full-length Yer067w, indicating that the overall structure remained intact (Figure S2). High-quality crystals of _11V-161N_Yer067w were obtained allowing the determination of structure to 1.7 Å resolution using the multi-wavelength anomalous dispersion (MAD) method. Data collection and refinement statistics for _11V-161N_Yer067w are summarized in Table 1.

**Table 1:** Data collection and refinement statistics.

Data collection				
Space group	P4_3_2_1_2			
Cell dimensions				
*a*, *b*, *c* (Å)	41.2, 41.2, 184.4			
	*Peak*	*Inflection*	*High Remote*	*Low Remote*
Wavelength	0.9790	0.9794	0.9642	0.9951
Resolution (Å)	50–1.61 (1.67–1.61)^a^	50–1.60 (1.66–1.60)	50–1.60 (1.66–1.60)	50–1.60 (1.66–1.60)
*R*_merge_	0.066 (0.112)	0.063 (0.106)	0.063 (0.117)	0.068 (0.150)
*I*/σ*I*	32.6 (7.4)	32.2 (7.1)	32.2 (6.9)	31.1 (4.5)
Completeness (%)	92.3 (55.4)	91.3 (50.9)	93.5 (60.6)	88.9 (40.7)
Redundancy	6.5 (1.7)	6.4 (1.6)	6.6 (1.8)	6.4 (1.5)

Refinement				
Resolution (Å)	19.5–1.70			
No. reflections	17,490			
*R*_work_/*R*_free_	0.196/0.238			
No. atoms				
Protein	1221			
Water	155			
*B*-factors				
Protein	7.4			
Water	21.2			
R.m.s deviations				
Bond lengths (Å)	0.013			
Bond angles (°)	1.37			
Ramachandran plot % of residues in most favorable regions	90.2			
Additional allowed regions	9.8			

a Highest resolution shell is shown in parenthesis.

**Figure 6:**
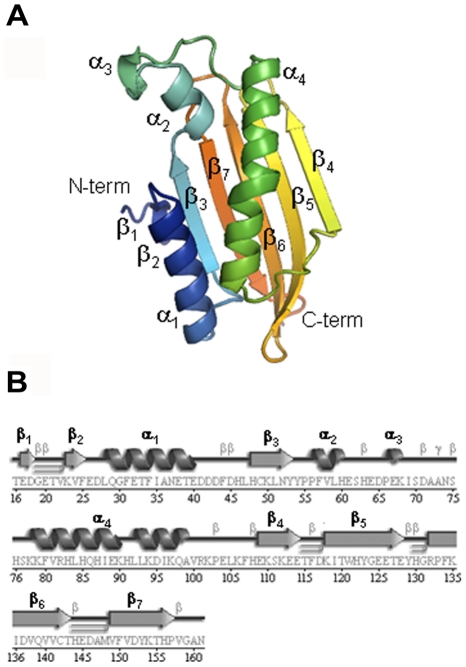
Crystal structure of _11V-161N_Yer067w protein. (A) Ribbon diagram of Yer067w color-coded from the N-terminus (blue) to C-terminus (red). Alpha-helices (α1-α4) and beta-strands (β1-β7) are labeled. (B) Secondary structure elements in Yer067w superimposed on its primary sequence. Beta and gamma-turns are denoted as β and γ, respectively. Beta-hairpins are shown as ⊃. The secondary structure diagram was generated by PDBsum.

**Figure 7:**
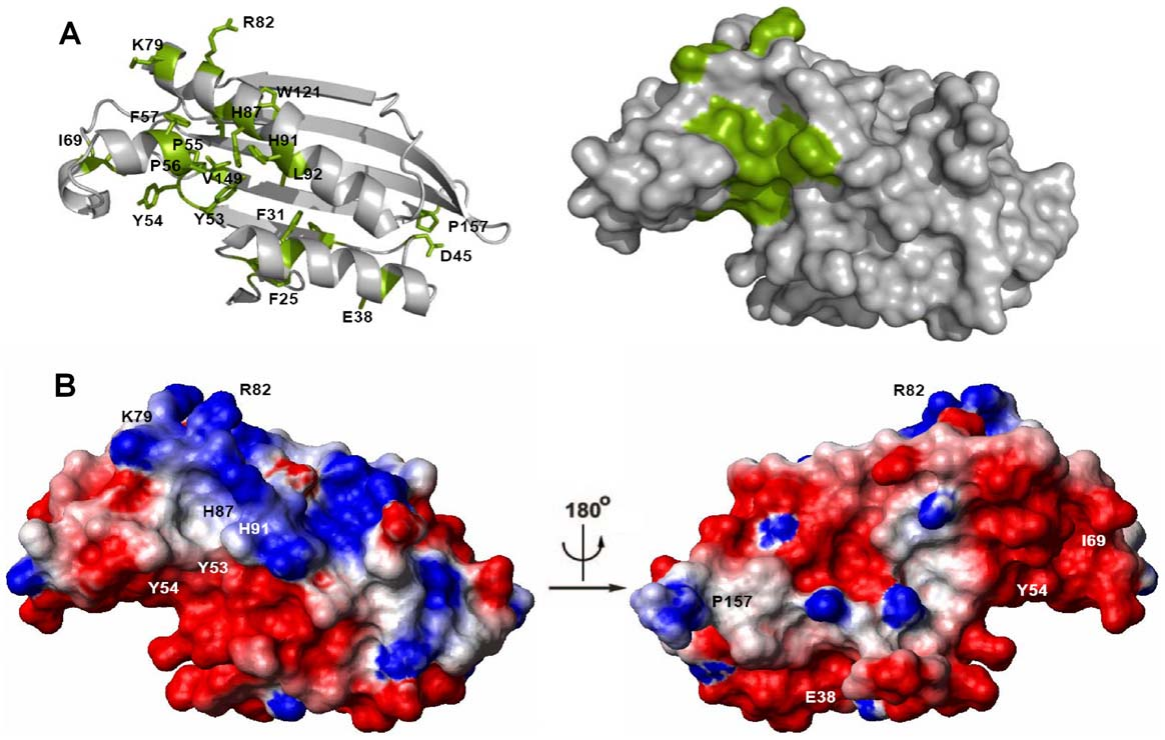
Position of the invariant residues of the Yer067w family in the three-dimensional structure. (A) Strictly conserved residues shown in Figure 1 are depicted in green with the respective lateral chain in the ribbon and space-filling diagram of Yer067w. Both diagrams are shown in the same orientation. Note the presence of a conserved cleft in Yer067w protein family. (B) Electrostatic surface charge distribution at pH 7.5 and 150 mM NaCl was calculated by Molmol. The positive and negative regions are shown in blue and red, respectively. The conserved, solvent-exposed residues are labeled. The first view is in the same orientation as shown in (A), while the second one shows the protein rotated through 180° along the vertical axis.

The structure reveals an α/β topology with a seven-stranded mixed β-sheet backed by four α-helices on one side (Figure 6A). The overall structure is compact and relatively rigid, in which the highest B-factor values, which reflect the fluctuation of atoms about their average positions in crystal structures, correspond to the N-terminal residues including the β1 strand (E17-D18). Notably, the first five residues of the construct (11VQTVT15) are disordered in the crystal. The secondary structure elements are arranged in the β1-β2-α1-β3-α2-α3-α4-β4-β5-β6-β7 order (Figure 6B). The slightly twisted β-sheet is formed by antiparallel strands, except for the β2-β3 pair, which is parallel. The short β2 strand connects to β3 through the helix α1, while β3 is followed by the cluster of helices α2, α3 and α4 before giving rise to the strand β4. The rest of the β-sheet is connected by short β-turns. The amphipathic helices α1 (L28-E40) and α4 (S77-V100) pack against the β-sheet, while the short α2 and α3 helices are projected outwards from the β-sheet surface. The longest helix, α4, transversally crosses the β-sheet and is mainly stabilized by hydrophobic interactions involving the nonpolar amino acids F80, L84, I88, L92, I96, and V100 that are facing the inner face of the β-sheet. Interestingly, this helix contains an unusual bulge in the fourth turn. The origin of this bulge is probably induced by the shift in hydrophobic periodicity, such that the E89-K90-H91 stretch is sandwiched between the H87-I88 and L92-L93 residues in the hydrophobic core. The side chain of H87 is stabilized in the hydrophobic core by a salt bridge with the side chain of Y53. The bulge is partially stabilized by a hydrogen bond between carbonyl of K90 and the side chain of K94 via an ordered water molecule. The surface of the protein is abundant in negatively charged residues (Figure 7B). The main exception is the patch of positively charged residues centered on the helix α4.

Analysis of crystal packing reveals that Yer067w forms a dimer in the crystal. The strands β4 of each monomer align into an antiparallel β-sheet to yield a continuous fourteen-strand β-sheet in the Yer067w dimer. The protein is unlikely to form a stable dimer in solution because the dimeric arrangement is not stabilized by any other intermolecular interactions besides the backbone hydrogen bonds. The biological relevance, if any, of the crystallization-produced Yer067w dimer remains to be seen.

To identify potential functional sites of Yer067w, we analyzed the location of residues conserved throughout the Yer067w family in the Yer067w structure (Figure 7A). Some of the strictly conserved Yer067w residues (F25, F31, F57, I69, L84, L92, W121, and V149) are buried in the structure and are important for structural stability. The most prominent patch of invariant surface residues is located in a cavity at the junction of all four helices (Figure 7A). It includes the invariant 53YYPPF57 stretch, which may be important to keep the shape of the cavity intact. The surface also contains two invariant histidines H87 and H91, amino acids that are frequently encountered in active sites of various enzymes [38]. However, these histidines are unlikely to be involved in catalytic activity because they are not neighbored by glutamates/aspartates that usually contribute to acid-base enzymatic mechanisms. However, the conserved cavity is long and has a largely hydrophobic character, hinting at the large size of the ligand and suggesting that this cavity is likely to be a protein-binding site. Even though not a part of the structured domain, the mobile N-terminus is positively charged due to a presence of multiple lysine residues and could present an interaction site with phospholipids.

**Figure 8:**
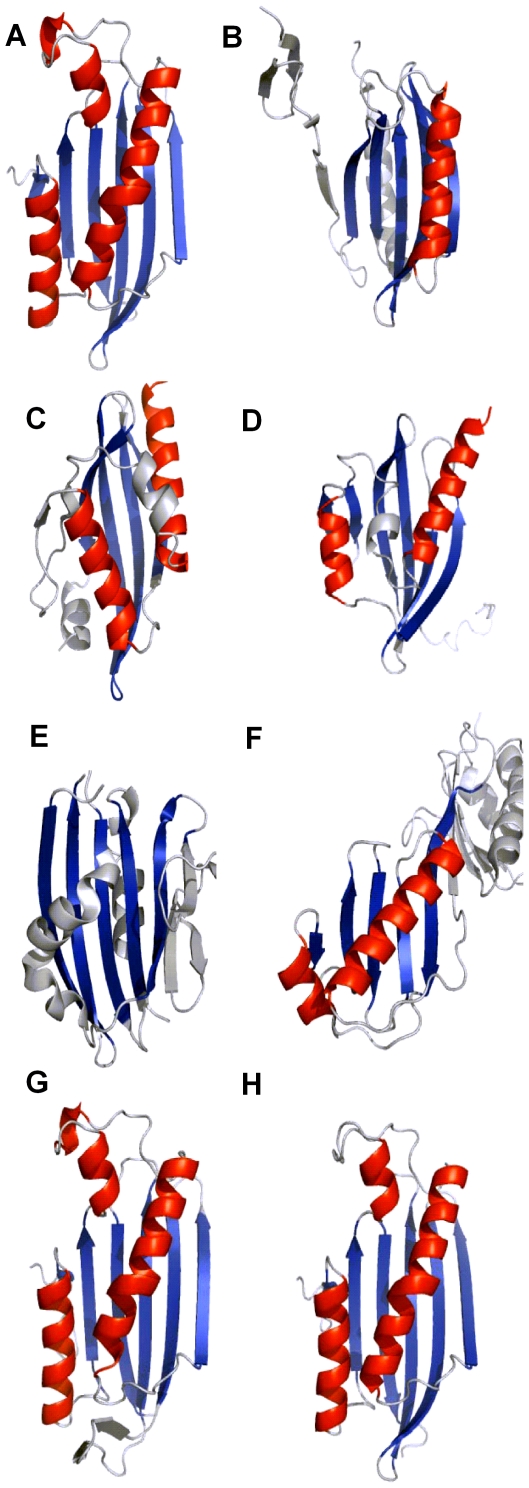
Protein structures that share some similarity to Yer067w according to DALI and SSM searches. The partially aligned β-strands and α-helices are shown in blue and red, respectively. Insertions are shown in gray. Note that most of the superimpositions are driven by the β-strands. Protein description and homology scores are as follows: (A)_11V-161N_Yer067w structure, PDB: 3bcy; (B) Unknown function protein PA94 from *Pseudomonas aeruginosa,* PDB: 1tu1, Z score: 5.9, Q score: 0.17; (C) NTF-like Bal32a from a Soil-Derived Mobile Gene Cassette, PDB: 1tuh, Z score: 3.2, Q score: 0.12; (D) NTF2-like protein of unknown function from *Burkholderia xenovorans,* PDB: 3en8, Z score: 4.3, Q score: 0.16, (E) PsbP protein in the Oxygen-Evolving Complex of Photosystem II from higher plants, PDB: 1v2b, Z score: 5.7, Q score: 0.11; (F) Adenovirus major late promoter TATA box-binding protein, PDB: 1qna, Z score: 1.8, Qscore: 0.041. For comparison, molecular modeling structures of Yer067w homologs are also shown: (G) DEHA0D4532 model, Z score: 26.2, Q score: 0.87. (H) Yil057c model, Z score: 28.5, Q score: 1.

To gather more clues about Yer067w's function, we searched for structural homologs of Yer067w using the DALI [39] and SSM [40] programs. Yer067w displays an overall two-layer α/β fold that is quite common, corresponding to the second most populated fold group among α/β proteins [41]. Nevertheless, both programs indicated that Yer067w shares only weak structural homology with a number of α/β structures (Figure 8 B). The best hit corresponds to a protein of unknown function from *Pseudomonas aeruginosa* (PDB ID code 1tu1), presenting a DALI Z-score of 5.9 (Z-scores below 2 are structurally dissimilar) and SSM Q-score of 0.17 (Q  = 1 means exact structural alignment) (Figure 8B). Despite the low scores obtained for these putative structural homologs, each of the matches was analyzed to identify key residues or structural motifs resembling the conserved core of the Yer067w family. However, the structural superpositions were driven almost exclusively by similarities among β-sheets, showing no resemblance to the conserved core around the junction of α-helices that is characteristic of the Yer067w family (Figure 8). Additionally, the Yer067w structure was refractory to functional predictions of the ProFunc server [42], despite the combination of different functional assignment approaches that included identification of smaller sub-motifs (e.g., helix-turn-helix, DNA binding patterns) and highly specific n-residue template methods (enzyme active sites, binding sites, DNA-binding residues and reverse template analysis).

The Yer067w fold could not be automatically classified into any of the α/β architectures/topologies using the program CATHEDRAL, as all matches showed sequence identity below 35% and an SSAP score below 80 [43]. The best scores (SSAP ranging from 70–74 and identity <10%) suggested several possible classifications, including Roll architecture, as in Nuclear transport factor (NTF)-like proteins (Figure 8 C, D) and a two-layer sandwich architecture (Figure 8F). The Yer067w structure differs from the NTF fold in two main aspects. While the NTF fold is characterized by a highly curved β-sheet flanked by α-helices forming a conical structure [44], the Yer067w β-sheet is only slightly twisted, forming a supporting surface for the α-helices rather than a roll-like structure. Furthermore, the C-terminal helix α4 of Yer067w is structurally aligned to the N-terminal helix α1 of NTF-like proteins, underlining the distinct topology among these proteins. The other possible classification is a two-layer sandwich architecture represented by the TATA-binding protein (PDB code 1qna), which agrees well with the overall fold of Yer067w: a β-sheet backed by α-helices on one side. However, we could not find any structures in the CATH database with the same order and configuration of secondary structure elements as those of Yer067w, precluding a straightforward classification in any of the topologies already classified as the two-layer sandwich architecture. Thus, the Yer067w structure likely defines a new subfamily of the α/β proteins, contributing to our understanding of the protein-fold space and the relationship between sequence and three-dimensional structure.

To complete the structural characterization of the Yer067w family, we modeled the three-dimensional structures of selected representatives from distinct phylogenetic branches using the program SwissModel with the Yer067w structure as a template (Figure 8G, H). The model of DEHA0D04532 from *Debaromyces hansenii*, the most distant Yer067w homolog, is highly similar to the Yer067w, demonstrating a Q-score of 0.87 and an RMSD of 0.3 Å, as calculated by SSM pairwise comparison (Figure 8G). The most obvious difference in the DEHA0D04532 model is an insertion between strands β5 and β6 that is predicted to form two short β-strands in the model. The structural models of the proteins from the post-WGD species are highly similar to the Yer067w structure, which reflects the high sequence identity between these proteins. For example, the structural model of Yil057c is nearly identical to that of Yer067w, with a Q-score of 1 and an RMSD of 0.06 Å (Figure 8H). Similar results were obtained for homologs of *Ashbya* or *Candida* (data not shown). These results suggest that the three-dimensional structure of the Yer067w family has undergone very minor changes over almost 170 million years, when the CTG group diverged from the Saccharomycotina complex [22]. These data point to a conserved functional role for this protein that was well-preserved during evolution.

Our phylogenetic analysis suggests that *YER067W* belongs to a class of genes that are particularly interesting for studying the genetics of evolutionary divergence, the slowly evolving orphan genes. These genes can be viewed as signatures of genetic pathways that have been newly acquired in a particular lineage and are of special importance for the respective lineage [45].

Additionally, the functional evidence points to an involvement of Yer067w in the oxidative activity of yeast that is coupled to ergosterol metabolism. The decrease in both parameters is associated with drug resistance, a phenomenon that is of both economical and medical interest, considering the presence of Yer067w homologs in pathogenic yeasts such as *Candida albicans* and *Candida glabrata*.

More than adding new information to the *YER067W*/*RGI1* Gene Ontology, we also present the first crystal structure for this new gene family of novel regulatory elements of aerobic energy metabolism in *Saccharomyces cerevisiae* and, probably, in other fungi species.

## Materials and Methods

### Alignment and phylogenetic analysis

The NCBI nr dataset was queried by BLAST and PSIBLAST searches using the Yer067w protein sequence NP_010990.1. Other fungal homologs were retrieved from the Orthogroups Repository [17]. Sequences were aligned using MUSCLE [46], and the figure was generated using JalView 2.4 [47].

### Yeast strains

The following *S. cerevisiae* strains were used. BYYer067w-TAP and BYYil057c-TAP containing the TAP tag introduced by homologous recombination at the down-stream portion of the gene. Deletion mutants BYΔyer067w (*MAT* ***a*** *his3 leu2 met15 ura3 yer067w::KANMX4),* BYΔyil057c (*MAT* ***a*** *his3 leu2 met15 ura3 yil057c::KANMX4)* and the isogenic strain BY4741 (*MAT* ***a*** *his3 leu2 met15 ura3*) were obtained from Open Biosystems.

A double mutant for *YER067W* and *YIL057C* was generated as described by Tong et al. [14], with minor modifications. Briefly, the original selection marker KANMX4 (conferring G418 resistance) of the strain BYΔyil057c was exchanged with NATMX4 for Nourseothricin (NAT) by homologous recombination upon transformation with the plasmid pCRII-TOPO::MX4-natR. The resultant strain BYΔyil057c::NATMX4 MAT**a** was crossed with the strain Y8205α (*MAT* ***α*** *his3 leu2 met15 ura3 can1Δ::STE2pr-HIS5 lyp1Δ::STE3pr-LEU2*). After sporulation, haploids Matα harboring yil057c::NATMX4 mutations were selected by the ability to grow in YPD-NAT and in synthetic media lacking leucine. The resulting haploid strain BYΔyer057c MAT**α** was crossed to strain BYΔyer067w MAT**a** (Open Biosystems). Sporulation of resultant diploid cells, followed by selection in media lacking histidine, led to the isolation of the haploid strain BYΔyer067wΔyil057c MAT**a**, harboring both the KANMX4 and NATMX resistance cassettes. The correct insertion of the selection markers was confirmed by PCR.

### Western blotting

Cells were grown either in complete rich medium YPD consisting of 1% yeast extract, 2% bactopeptone, supplemented with 2% D-glucose or 2% glycerol (YPG) at 28°C. For heat shock assay, cells grown in YPD were subjected to 37°C for one hour. At the end of the treatments, OD_600nm_ was determined, and the 3 units of absorbance were collected by centrifugation. Yeast cells were resuspended in gel-loading buffer with protease inhibitor cocktail (Roche) and submitted to two cycles of freezing/thawing in N_2_ liquid followed by glass beads vortexing. Total protein extract was resolved by SDS–polyacrylamide gel electrophoresis (15% polyacrylamide) and electroblotted from the gel onto a PVDF membrane using Tris–glycine buffer and then blocked overnight in 5% (w/v) skim milk solution. The blot was probed with the anti-TAP polyclonal antibody diluted 1:5000 (Open Biosystems), followed by incubation with peroxidase-conjugated 1∶5000 diluted antibody (Amersham, GE). The signal was detected using the ECL Plus Western Detection Kit (Amersham, GE).

### Plate growth assay

BY4741 (WT), BYΔyer067w, BYΔyer067wΔyil057c were grown in YPD up to stationary phase. Cells were normalized at a concentration of 10^7^ cells.mL^−1^ (OD_600nm_  = 1.0) and serially diluted 3 times, 10-fold each step. Using a replica plate, the serial dilutions of the suspension were patched in YP plates supplemented with 2% glucose, 2% galactose, 2% ethanol or 2% glycerol. Plates were incubated at 30°C or 37°C for 2 days and then photographed. All plates were made in duplicate for each treatment, and the experiment was repeated three times.

### Intracellular glycogen and trehalose quantification

The strains BY4741, ByΔYer067w, ByΔYil057c, ByΔYer067wΔYil057c were grown in medium YPD at 28°C until the first log phase or stationary phase. The intracellular content of glycogen and trehalose was determined as previously described [48]. Briefly, 50 units of absorbance (OD_600nm_) of yeast cells were centrifuged and then washed two times with 1 mL of cold water. The pellet was resuspended in 500 µL of 0.25 M Na_2_CO_3_ and boiled for one hour for total carbohydrate extraction. Glycogen was digested by incubating 160 µL of the suspension, previously neutralized using 40 µL of 3 M acetic acid, with 8 µL amiloglucosidase (Fluka, 75 U/mL) in 560 µL of 0.2 M sodium acetate (pH 5.2). For trehalose determination, 40 µL of the suspension were neutralized with 10 µL of 3 M acetic acid, and 1 µL of trehalase (Sigma 3.7 U/mL) was added along with 140 µL of 0.2 M sodium acetate (pH 5.2). Reactions were kept at 50°C and 37°C overnight for glycogen and trehalose digestion, respectively. Samples were then boiled for 5 minutes to inactivate the enzymes and centrifuged. The glucose content of 25 µL of supernatant was assayed by adding 200 µL of glucose oxidase mixture (Glucox 500 - DOLES) and read at 510 nm in a microplate reader apparatus. The experiment was repeated three times.

### Drug susceptibility assay

Qualitative data were generated as described in the “plate growth assay” section above, with the exception that cells were patched in YPD plates containing 16.0 µg/mL of fluconazol or 1.0 µg/mL of amphotericin B. For the quantitative assays, cells were grown to the stationary phase in YPD medium and then diluted to standardize the inoculums to around 1,000 cells for each well of the 96-well microplates. Fluconazole and amphotericin B were diluted in YPD to obtain the final working solution (2x concentrated), which was serially diluted two times for each step, leaving 100 µL of media in each well. After that, 100 µL of cell suspension were added, and the cultures were allowed to grow at 28°C with agitation for 16 hours. Optical density at 600 nm was determined using a microplate reader. The percentage of growth relative to the drug-free control was calculated for each strain. Experiments were independently repeated two times in triplicate for each data point. Standard errors are smaller than the graph symbols.

### Isolation of total sterols and gas mass chromatography analysis

BY4741 and BYΔyer067w yeast cells were grown overnight at 28°C in YPD medium. Briefly, 150 units of absorbance (OD_600nm_) of yeast cells were harvested by centrifugation and washed two times with 1 mL of cold water, 10 µg of cholesterol (Sigma) was added to the pellet as an exogenous extraction control. Total lipids were extracted according Schneiter and Daum [49] and dried under a stream of nitrogen. Total sterol content was extracted by saponification [50]. After drying in nitrogen, sterols were resuspended in 50 µL silylant STFA, TMCS 99∶1 (Sigma-Aldrich) + 50 µL pyridine followed by incubation for one hour at 65°C. GC/MS analysis was carried out on a Shimadzu GCMS-QP2010 Plus system, using an Rtx®-5MS (5% phenyl 95% dimethylpolysiloxane), of Restek® (30 m×0.25 mm×0.25 µm). The injector was set at 250°C. The column temperature was programmed from 170–250°C at 20°C/min, 250–280°C at 5°C/min and held at 280°C for 20 min. Helium was used as the carrier gas with a linear velocity of 41.9 cm s^−1^. A volume of 1 µL of sample was injected into the chromatograph. Electroionization (EI-70 eV) and a quadrupole mass analyzer operated in scans from 50 to 700 amu. The interface was set at 230°C and the ion source at 200°C. The components were identified by comparing their mass spectra with those of the library NIST05 contained in the computer's mass spectrometer. Retention indices were also used to confirm the identity of the peaks in the chromatogram.

### Whole cells respiratory activity

Oxygen consumption by *Saccharomyces cerevisiae* (1×10^6^ cells ml^−1^ grown in synthetic media supplemented with 3% glycerol) was measured at 28°C using a high-resolution oxygraphic system (Oroboros Oxygraph-O2K). The electrode was calibrated between 0 and 100% saturation with atmospheric oxygen, and respiratory rates were measured under normoxic conditions. The relative changes in oxygen consumption rate were determined after addition of 5 µg/ml of oligomycin and 1.0 µM of carbonyl cyanide *p*-trifluoromethoxyphenylhydrazone (FCCP). Experiments were independently repeated three times for each data point.

### Protein expression, preparation and purification

Residues 11-161 of *S. cerevisiae* Yer067w were cloned into the pET15b vector (Amersham-Pharmacia) and expressed in *E. coli* BL21(DE3) in rich (LB) medium as a N-terminal His-tag fusion. For production of a selenomethionine-labeled protein, the expression plasmid was transformed into the *E. coli* methionine auxotroph strain DL41(DE3), and the protein was produced using LeMaster medium [51]. Cells were harvested and lysed in 50 mM HEPES (pH 7.4), 300 mM NaCl, 1% (v/v) β-mercaptoethanol and 5% glycerol. The fusion protein was purified by affinity chromatography on Ni^2+^-charged chelating sepharose resin, and the tag was removed by cleavage with thrombin, leaving a Gly-Ser-His-Met N-terminal extension. The cleaved protein was additionally purified using size-exclusion chromatography using HPLC buffer (10 mM HEPES, 50 mM NaCl, 1 mM DTT, pH 7.0). The selenomethionine-labeled protein was purified in a similar manner.

### Anti-Yer067w specific antibody

Two rabbits were immunized with purified recombinant full-length protein Yer067w (0.12 mg) emulsified in complete Freud's adjuvant (Sigma) and injected subcutaneously at multiple sites on their backs. Two weeks later, this procedure was repeated using incomplete Freud's adjuvant (Sigma). The immunization program was completed with an intramuscular injection. The serum from rabbits bled one month after the last booster was checked against the Yer067w antigen by dot blot analysis.

### Yer067w localization analysis by subcellular fractionation and immunofluorescence microscopy

Yeast cells BY4741 grown in YP glycerol were collected by centrifugation and washed twice with cold water. The cell pellets were suspended in 500 µL SEM buffer (20 mM MES, 1 mM PMSF, 1 mM EGTA, 2 mM MgCl_2_, 0.6 M Sorbitol, pH 6.0) supplemented with protease inhibitors cocktail (10 mg/mL each of pepstatin, leupeptin, aprotinin and 1 mM PMSF). Cells were disrupted using glass beads by vortexing in 10 bursts over 1 min and cooling on ice between bursts. The extract was clarified by centrifugation at 1000 g during 10 minutes at 4°C. A 100 µL sample of the total protein extract was collected (Tot), and the remaining 400 µL was centrifuged at 12,000 g for 20 minutes at 4°C. The resulting pellet was suspended in 400 µL SEM (P1), and the supernatant further centrifuged at 100,000 g for 40 minutes at 4°C. The 100,000 g pellet, enriched in plasma membrane-bound proteins, was suspended in 400 µL SEM (P2), and the supernatant was collected as the soluble protein fraction (Snt). The same volume of each fractioning step was used for western blot analysis, performed as described above. The following antibodies were used: anti-Yer067w 1∶2,000 (this work), anti-Act1 1∶500 (Santa Cruz Biotechnology), anti-Pma1 and anti-Pdr5 1∶10,000 (kindly provided by Dr. Michel Ghislain, Université Catholique Louvain la Neuve).

Yer067w localization by indirect immunofluorescence microscopy was performed as described [52]. Anti Yer067w 1∶50 serum was pre-adsorbed in 1% BYΔyer067w acetone powder and incubated for 2 hours with the fixed BY4741 and BYΔyer067w cells. ALEXA fluor 488-conjugated anti-rabbit (Molecular Probes) was diluted 1∶1000 and incubated for one hour. Fluorescent labeling was analyzed on an Axiophot microscope (Zeiss).

### _11V-161N_Yer067w Crystallization

Initial crystallization conditions were identified by means of hanging drop vapor diffusion using sparse matrix screens (QIAGEN). The best crystals were obtained by equilibrating a 1.5 µL drop of a protein (6 mg/mL) in buffer (10 mM HEPES, 50 mM NaCl, 1 mM DTT, pH 7.0), mixed with 1.5 µL of reservoir solution containing 13% (w/v) PEG 6000, 0.2 M ammonium chloride, 20% glycerol, and 0.1 M sodium acetate (pH 5.0) and suspended over 1 mL of reservoir solution. Crystals grew over 3–14 days at 20°C. For data collection, crystals were picked up in a nylon loop and flash cooled in an N_2_ cold stream (Oxford Cryosystem). The crystals contained one molecule in the asymmetric unit (Z  = 8) corresponding to V_m_  = 2.15 Å^3^ Da^−1^ and a solvent content of 42.8% [53].

### _11V-161N_Yer067w structure solution and refinement

Diffraction data from a SeMet-labeled crystal of Yer067w were collected using a four-wavelength MAD regime on an ADSC Quantum-210 CCD detector (Area Detector Systems Corp.) at beamline F2 at the Cornell High-Energy Synchrotron Source (CHESS) (Table 1). Data processing and scaling were performed with HKL2000 [54]. The structure was determined by MAD phasing using the program SHELX [55]. Density modification with the program ARP/wARP [56] allowed for automated model building of >90% of the residues.

The partial model obtained from ARP/wARP was extended manually with the help of the program Xfit [57] and was improved by several cycles of refinement, using the program REFMAC [58] and model refitting, followed by the translation-libration-screw (TLS) refinement [59]. Out of 155 residues of the construct, the final model does not include the 9 N-terminal residues including the GSHM cloning artifact. In addition, 155 water molecules were included in the model. The final model has good stereochemistry, with no outliers in the Ramachandran plot computed using PROCHECK [60]. Coordinates have been deposited in the RCSB Protein Data Bank with accession code 3bcy.

### Molecular modeling of Yer067w protein sequence homologs

Tridimensional models of Yil057c and DEHA0D04532 were generated by the program SwissModel using Yer067w structure as the template [61]. The final energy for the Yil057c and DEHA0D04532 model was −5826.193 kJ/mol and −5206.979 kJ/mol, respectively.

## Supporting Information

Figure S1 Phylogenetic analysis of Yer067w protein family. The evolutionary profile of Yer067w family was inferred using the Neighbor-Joining method, and the bootstrap consensus tree was generated from 500 replicates (Saitou et al, 1987). Evolutionary distances were computed using the Poisson correction method. All positions containing gaps and missing data were eliminated from the dataset (Complete deletion option). There were a total of 155 positions in the final dataset. Phylogenetic analysis was conducted in MEGA4 (Tamura et al, 2007).The percentage of replicate trees in which the associated taxa clustered together in the bootstrap test is shown next to the branches. The tree is drawn to scale, with branch lengths in the same units as those of the evolutionary distances used to infer the phylogenetic tree. Cluster (a) contains organisms that translate GTC as serine instead of leucine. Cluster (b) is formed by proteins from species that diverged before (branch c) and after the whole genome duplication event. The species and gene notation follows the code described in Fig. 1.
(2.86 MB TIF)

Figure S2 Overlay of the 2D [15N,1H]-HSQC spectra of the 15N-labeled full-length Yer067w (cyan contours) and the truncated _11V161N_Yer067w (red contours). Almost all dispersed peaks of _11V161N_Yer067w are perfectly superposed to the full-length protein, a strong indication that the overall fold is preserved in the shortened version.
(0.05 MB PDF)

Table S1 Genes that share a similar transcriptional expression pattern to *YER067W* and *YIL057C*. Summary of the results retrieved from the program SPELL (http://imperio.princeton.edu:3000/yeast). This program identifies which microarray datasets are most informative for the query gene. Genes with expression profiles similar to the query are identified within these datasets. The *YER067W* and *YIL057C* expression profiles were compared against approximately 100 microarray experiments.
(0.03 MB PDF)

Table S2 Transcriptional factor binding motifs present in *YER067W* and *YIL057C* promoters. These data refer to the predicted putative binding sites with indirect or direct experimental evidence. Data were generated by the YeastStract server (http://www.yeastract.com).
(0.04 MB PDF)
