# Supplementary figures and images for: Structural and Functional Study of Yer067w, a New Protein Involved in Yeast Metabolism Control and Drug Resistance
Source: PLoS One. 2010 Jun 17;5(6):e11163. doi: 10.1371/journal.pone.0011163 (PMC2887356; doi:10.1371/journal.pone.0011163)

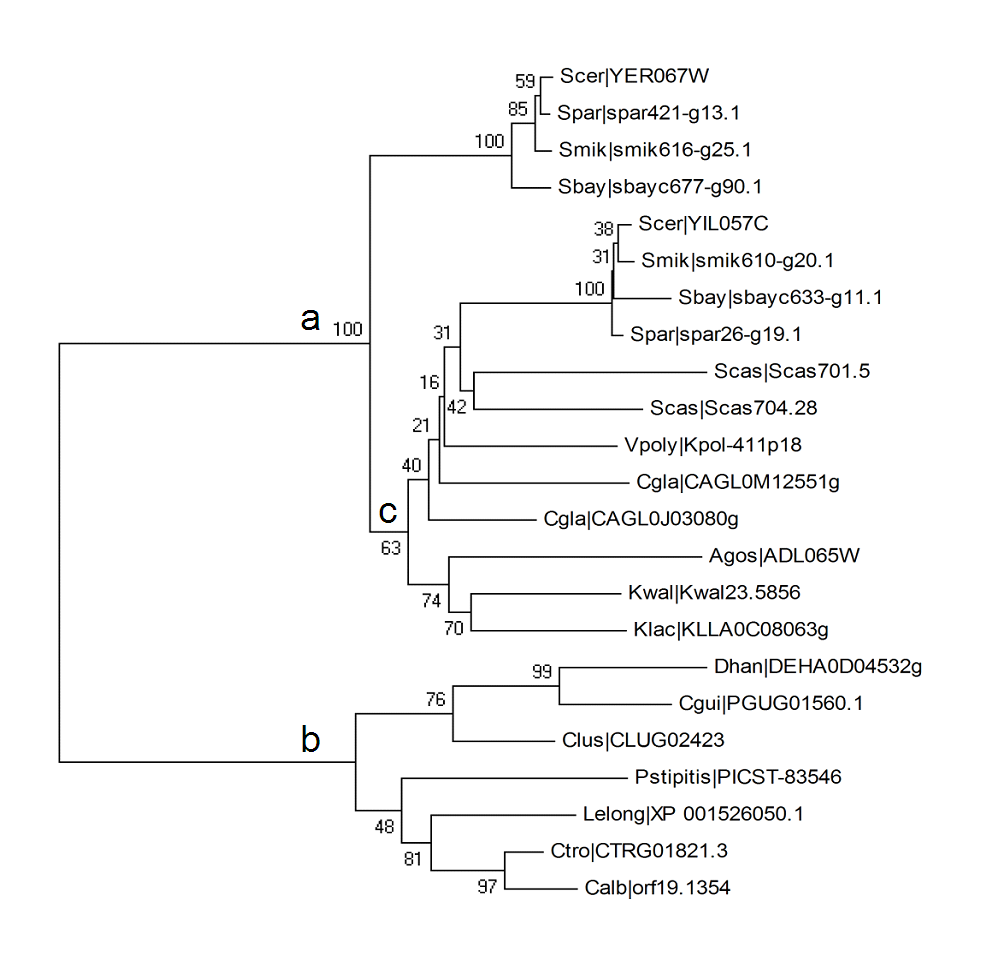

Supplement: Figure S1 — Phylogenetic analysis of Yer067w protein family. The evolutionary profile of Yer067w family was inferred using the Neighbor-Joining method, and the bootstrap consensus tree was generated from 500 replicates (Saitou et al, 1987). Evolutionary distances were computed using the Poisson correction method. All positions containing gaps and missing data were eliminated from the dataset (Complete deletion option). There were a total of 155 positions in the final dataset. Phylogenetic analysis was conducted in MEGA4 (Tamura et al, 2007).The percentage of replicate trees in which the associated taxa clustered together in the bootstrap test is shown next to the branches. The tree is drawn to scale, with branch lengths in the same units as those of the evolutionary distances used to infer the phylogenetic tree. Cluster (a) contains organisms that translate GTC as serine instead of leucine. Cluster (b) is formed by proteins from species that diverged before (branch c) and after the whole genome duplication event. The species and gene notation follows the code described in Fig. 1. (2.86 MB TIF) [file pone.0011163.s001.tif]

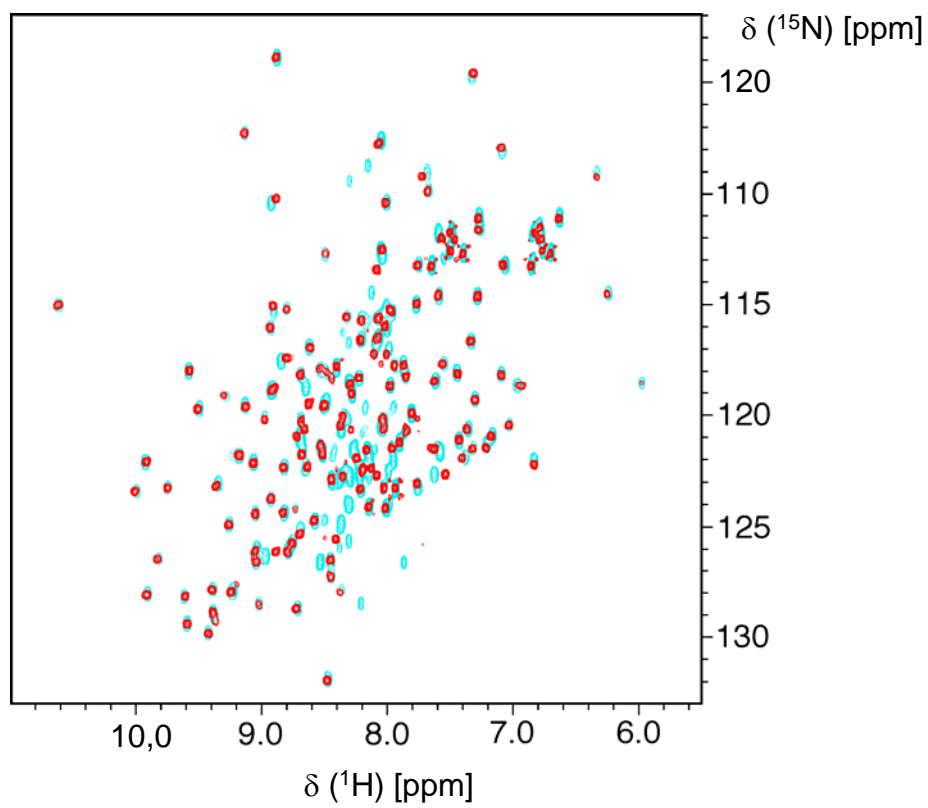

Supplement: Figure S2 — Overlay of the 2D [15N,1H]-HSQC spectra of the 15N-labeled full-length Yer067w (cyan contours) and the truncated 11V161NYer067w (red contours). Almost all dispersed peaks of 11V161NYer067w are perfectly superposed to the full-length protein, a strong indication that the overall fold is preserved in the shortened version. (0.05 MB PDF) [file pone.0011163.s002.pdf]
