# Supplementary material for: Structural and Functional Study of Yer067w, a New Protein Involved in Yeast Metabolism Control and Drug Resistance
Source: PLoS One. 2010 Jun 17;5(6):e11163. doi: 10.1371/journal.pone.0011163 (PMC2887356; doi:10.1371/journal.pone.0011163)
Supplement: Table S1 — Genes that share a similar transcriptional expression pattern to YER067W and YIL057C. Summary of the results retrieved from the program SPELL (http://imperio.princeton.edu:3000/yeast). This program identifies which microarray datasets are most informative for the query gene. Genes with expression profiles similar to the query are identified within these datasets. The YER067W and YIL057C expression profiles were compared against approximately 100 microarray experiments. (0.03 MB PDF) [file pone.0011163.s003.pdf]

**SPELL results for genes co-regulated with *YER067W***

| GOTerm | P-val | Annotated Genes |
|---|---|---|
| energy reserve metabolic process (biological_process) | 2.25e-05 | GSY2, GLC3, TSL1, GSY1, TPS2 |
| cellular carbohydrate metabolic process (biological_process) | 5.22e-05 | GSY2, PGM2, GLC3, TSL1, SOL4, HXK1, GSY1, TPS2 |
| carbohydrate metabolic process (biological_process) | 1.01e-04 | GSY2, PGM2, GLC3, TSL1, SOL4, HXK1, GSY1, TPS2 |
| carbohydrate biosynthetic process (biological_process) | 6.74e-04 | GSY2, GLC3, TSL1, GSY1, TPS2 |
| glycogen biosynthetic process (biological_process) | 1.85e-03 | GSY2, GLC3, GSY1 |
| UDP-glucosyltransferase activity (molecular_function) | 2.39e-03 | GSY2, TSL1, GSY1 |
| glycogen (starch) synthase activity (molecular_function) | 2.87e-03 | GSY2, GSY1 |
| generation of precursor metabolites and energy (biological_process) | 3.61e-03 | STF2, GSY2, GLC3, TSL1, GSY1, TPS2 |
| glucosyltransferase activity (molecular_function) | 3.79e-03 | GSY2, TSL1, GSY1 |
| trehalose-phosphatase activity (molecular_function) | 8.59e-03 | TSL1, TPS2 |
| glucan biosynthetic process (biological_process) | 9.40e-03 | GSY2, GLC3, GSY1 |
| UDP-glycosyltransferase activity (molecular_function) | 1.66e-02 | GSY2, TSL1, GSY1 |
| alpha,alpha-trehalose-phosphate synthase complex (UDP-forming) (cellular_component) | 1.72e-02 | TSL1, TPS2 |
| trehalose biosynthetic process (biological_process) | 1.72e-02 | TSL1, TPS2 |
| disaccharide biosynthetic process (biological_process) | 1.72e-02 | TSL1, TPS2 |
| energy derivation by oxidation of organic compounds (biological_process) | 1.75e-02 | GSY2, GLC3, TSL1, GSY1, TPS2 |
| transferase activity, transferring hexosyl groups (molecular_function) | 2.79e-02 | GSY2, GLC3, TSL1, GSY1 |
| glycogen metabolic process (biological_process) | 2.96e-02 | GSY2, GLC3, GSY1 |
| polysaccharide biosynthetic process (biological_process) | 4.38e-02 | GSY2, GLC3, GSY1 |

**SPELL results for genes co-regulated with *YIL057C***

| GOTerm | P-val | Annotated Genes |
|---|---|---|
| peroxisome (cellular_component) | 5.95e-03 | PXA1, CTA1, POT1, ECI1 |
| microbody (cellular_component) | 5.95e-03 | PXA1, CTA1, POT1, ECI1 |
| ammonium transporter activity (molecular_function) | 3.45e-02 | ADY2, ATO2 |
| ammonium transport (biological_process) | 3.45e-02 | ADY2, ATO2 |
| organic cation transport (biological_process) | 3.45e-02 | ADY2, ATO2 |
| organic cation transporter activity (molecular_function) | 3.45e-02 | ADY2, ATO2 |
| microbody part (cellular_component) | 3.87e-02 | PXA1, CTA1, POT1 |
| peroxisomal part (cellular_component) | 3.87e-02 | PXA1, CTA1, POT1 |
