# Supplementary material for: Structural and Functional Study of Yer067w, a New Protein Involved in Yeast Metabolism Control and Drug Resistance
Source: PLoS One. 2010 Jun 17;5(6):e11163. doi: 10.1371/journal.pone.0011163 (PMC2887356; doi:10.1371/journal.pone.0011163)
Supplement: Table S2 — Transcriptional factor binding motifs present in YER067W and YIL057C promoters. These data refer to the predicted putative binding sites with indirect or direct experimental evidence. Data were generated by the YeastStract server (http://www.yeastract.com). (0.04 MB PDF) [file pone.0011163.s004.pdf]

Transcription Factor biding motifs in YER067W promoter	Functional Description	Evidence
	Energetic Metabolism	
Adr1p	Carbon source-responsive zinc-finger transcription factor, required for transcription of the glucose-repressed gene ADH2, of peroxisomal protein genes, and of genes required for ethanol, glycerol, and fatty acid utilization	(*Direct: ChIP-on-chip*) Workman CT et al., Science, 2006;312(5776):1054-59
Cat8p	derepression of a variety of genes under non-fermentative growth conditions, active after diauxic shift, binds carbon source responsive elements	(*Indirect: Microarrays - Wild type vs. TF mutant*) Young ET et al., J Biol Chem, 2003;78(28):26146-58
Hap4p	Subunit of the heme-activated, glucose-repressed Hap2p/3p/4p/5p CCAAT-binding complex, a transcriptional activator and global regulator of respiratory gene expression; provides the principal activation function of the complex	*Indirect: Microarrays - Wild type vs. TF mutant*) Lascaris R et al., Microbiology, 2004 Apr;150(0):929-34
Stp1/Stp2	Homologous transcription factors, activated by proteolytic processing in response to signals from the SPS sensor system for external amino acids; activates transcription of amino acid permease genes and may have a role in tRNA processing	(*Indirect: Microarrays - Wild type vs. TF mutant*) Eckert-Boulet N et al., Yeast, 2004;1(8):635-48
	Stress Response	

Hsf1p	Trimeric heat shock transcription factor, activates multiple genes in response to stresses	*(Indirect: Microarrays - Wild type vs. TF mutant*) Yamamoto et al., J Biol Chem., 2005 Mar 25;280(12):11911-9 (*Indirect: Proteome - Wild type vs. TF mutant*) Boy-Marcotte et al., Mol Microbiol, 1999; (2): 274-283.
Msn2/Msn4	Homologous zinc finger transcriptional factors activated in several stress conditions, which results in translocation from the cytoplasm to the nucleus.	*(Indirect: Microarrays - Wild type vs. TF mutant*) Gasch et al., Mol Biol Cell, 2000; (11): 4241-4257. (*Indirect: Proteome - Wild type vs. TF mutant*) Boy-Marcotte et al., Mol Microbiol, 1999; (2): 274-283.
Yap1p	Basic leucine zipper (bZIP) transcription factor required for oxidative stress tolerance; activated by H2O2 through the multistep formation of disulfide bonds and transit from the cytoplasm to the nucleus; mediates resistance to cadmium	(*Direct: ChIP-on-chip*) Workman CT et al., Science, 2006;312(5776):1054-59 (*Indirect: Microarrays - Wild type vs. TF mutant*) Horan et al., Yeast, 2006;23(7):519-535 Thorsen et al., Physiol Genomics, 2007 Jun 19;30(1):35-43
	Other	
Fkh2p	Forkhead family transcription factor with a major role in the expression of G2/M phase genes; positively regulates transcriptional elongation; negative role in chromatin silencing at HML and HMR; substrate of the Cdc28p/Clb5p kinas	(*Direct: ChIP-on-chip*) Workman CT et al., Science, 2006;312(5776):1054-59 (*Direct: ChIP-on-chip*)
Aft1p	Transcription factor involved in iron utilization and homeostasis; activates	(*Indirect: Microarrays - Wild type vs. TF mutant*) Pagani et al., Mol

	the expression of target genes in response to changes in iron availability	Microbiol, 2007 Jul;65(1):521-537

Transcription Factor biding motifs in YIL057C promoter	Functional Description	Evidence
	Energetic Metabolism	
Adr1p	Carbon source-responsive zinc-finger transcription factor, required for transcription of the glucose-repressed gene ADH2, of peroxisomal protein genes, and of genes required for ethanol, glycerol, and fatty acid utilization	(*Direct: ChIP-on-chip*) Tachibana C et al., Mol Cell Biol, 2005;25(6):2138-46 (*Direct: ChIP*) Young ET et al., J Biol Chem, 2003;78(28):26146-58
Stp1/Stp2	Homologous transcription factors, activated by proteolytic processing in response to signals from the SPS sensor system for external amino acids; activates transcription of amino acid permease genes and may have a role in tRNA processing	(*Indirect: Microarrays - Wild type vs. TF mutant*) Eckert-Boulet N et al., Yeast, 2004;1(8):635-48
	Stress Response	
Arr1p	Transcriptional activator of the bZIP family, required for transcription of genes involved in resistance to arsenic compounds	(*Indirect: Microarrays - Wild type vs. TF mutant*) Haugen AC et al., Genome Biol, 2004;5(12):R95
Sfp1p	Transcription factor that controls expression of many ribosome biogenesis genes in response to nutrients and stress, regulates G2/M transitions during mitotic cell cycle and DNA-damage	(*Indirect: Microarrays - Wild type vs. TF mutant*) Cipollina et al, Microbiology, ;154(1):337-46

	response, involved in cell size modulation	
	Cell Cycle	
Fhl1p	Transcriptional activator with similarity to DNA-binding domain of Drosophila forkhead but unable to bind DNA in vitro; required for rRNA processing; isolated as a suppressor of splicing factor prp4	(*Direct: ChIP-on-chip*) Kasahara et al, Mol Cell Biol, ;27(19):6686-705
Rap1p	DNA-binding protein involved in either activation or repression of transcription, depending on binding site context; also binds telomere sequences and plays a role in telomeric position effect (silencing) and telomere structure	(*Direct: ChIP-on-chip*) Kasahara et al, Mol Cell Biol, ;27(1 9):6686-705
Xbp1p	Transcriptional repressor that binds to promoter sequences of the cyclin genes, CYS3, and SMF2; expression is induced by stress or starvation during mitosis, and late in meiosis; member of the Swi4p/Mbp1p family; potential Cdc28p substrate	(*Indirect: Microarrays - Wild type vs. TF mutant*) Chua G et al., PNAS, 2006;103(32):12045-50
Yhp1p	One of two homeobox transcriptional repressors (see also Yox1p), that bind to Mcm1p and to early cell cycle box (ECB) elements of cell cycle regulated genes, thereby restricting ECB-mediated transcription to the M/G1 interval	(*Indirect: Microarrays - Wild type vs. TF mutant)* Chua G et al., PNAS, 2006;103(32):12045-50
Yox1p	Homeodomain-containing transcriptional repressor, binds to Mcm1p and to	(*Indirect: Microarrays - Wild type vs. TF mutant)* Chua G et al., PNAS,

	early cell cycle boxes (ECBs) in the promoters of cell cycle-regulated genes expressed in M/G1 phase; expression is cell cycle-regulated; potential Cdc28p substrate	2006;103(32):12045-50
Tos8p	Homeodomain-containing transcription factor; SBF regulated target gene that in turn regulates expression of genes involved in G1/S phase events such as bud site selection, bud emergence and cell cycle progression; similarity to Cup9p	(*Indirect: Microarrays - Wild type vs. TF mutant*) Chua G et al., PNAS, 2006;103(32):12045-
	Pseudohyphal growth	
Flo8p	Transcription factor required for flocculation, diploid filamentous growth, and haploid invasive growth; genome reference strain S288C and most laboratory strains have a mutation in this gene	(*Direct: ChIP-on-chip*) Borneman AR et al., Genes and Development, 2006;20(4):435-448
Mga1p	Protein similar to heat shock transcription factor; multicopy suppressor of pseudohyphal growth defects of ammonium permease mutants	(*Direct: ChIP-on-chip*) Borneman AR et al., Genes and Development, 2006;20(4):435-448
Phd1p	Transcriptional activator that enhances pseudohyphal growth; regulates expression of FLO11, an adhesin required for pseudohyphal filament formation; similar to StuA, an A. nidulans developmental regulator; potential Cdc28p substrate	(*Direct: ChIP-on-chip*) Borneman AR et al., Genes and Development, 2006;20(4):435-448
Sok2p	Nuclear protein that plays a	(*Direct: ChIP-on-chip*)

	regulatory role in the cyclic AMP (cAMP)-dependent protein kinase (PKA) signal transduction pathway; negatively regulates pseudohyphal differentiation; homologous to several transcription factors	Borneman AR et al., Genes and Development, 2006;20(4):435-448
